# Evaluating the Effectiveness of Immersive Virtual Reality Rehabilitation Games With Enhanced Visual Training for Hand Motor Function Improvement Using Electromyography: Randomized Controlled Trial

**DOI:** 10.2196/74314

**Published:** 2025-11-25

**Authors:** Faisal Amin, Asim Waris, Muhammad Jawad Khan, Muhammad Adeel Ijaz, Hammad Nazeer, Syed Omer Gilani, Fawwaz Hazzazi, Umer Hameed Shah

**Affiliations:** 1 Department of Biomedical Engineering and Sciences, School of Mechanical and Manufacturing Engineering, National University of Sciences and Technology, NUST, H-12, Islamabad, 44000, Pakistan, 92 5190856068; 2 Department of Mechatronics Engineering, Air University, Islamabad, Pakistan; 3 Department of Electrical, Computer, and Biomedical Engineering, Abu Dhabi University, Abu Dhabi, United Arab Emirates; 4 Department of Electrical Engineering, College of Engineering, Prince Sattam bin Abdulaziz University, Al-Kharj, Saudi Arabia; 5 Department of Mechanical Engineering and Artificial Intelligence Research Center, College of Engineering and Information Technology, Ajman University, Ajman, United Arab Emirates

**Keywords:** electromyography, virtual reality, games, visual feedback, hand motor function

## Abstract

**Background:**

Hand motor dysfunction greatly reduces the performance of stroke survivors. This affects their ability to perform hand motor tasks effectively. Patients receive slow interventions due to interventional limitations in stroke rehabilitation, which can pose challenges for sustaining enduring improvements. We developed immersive virtual reality (VR) games that used an innovative approach to cognitive engagement within visual training feedback for achieving long-lasting improvements.

**Objective:**

This study aimed to evaluate the effectiveness of fully immersive VR-based hand games compared with conventional physical therapy and to assess the correlations between electromyographic data and clinical outcome measures for improving hand motor function in patients with subacute stroke.

**Methods:**

A randomized controlled study was conducted among 52 patients with subacute stroke who met the inclusion criteria. These patients were equally allocated to an experimental group (n=26) and a control group (n=26). The experimental group received both fully immersive VR-based hand game therapy and conventional physical therapy, whereas the control group received only conventional physical therapy. Owing to the nature of the intervention, the study was unblinded, and both therapists and patients were aware of the intervention. Both groups participated in intervention sessions 4 days a week for 6 weeks (24 sessions in total). Moreover, both groups underwent 2 weeks of follow-up. Clinical outcome measures, including the Fugl-Meyer Assessment-upper extremity (FMA-UE), Action Research Arm Test (ARAT), and Box and Block Test (BBT), were used to assess motor recovery and functional performance. The minimal clinically meaningful difference (MCID) was used for comparing clinical outcome measures to examine clinically meaningful improvements. Furthermore, the correlation between electromyography data and clinical outcome measures, and the weekly progression in movement performance were evaluated to identify improvements in hand motor function.

**Results:**

After the intervention, there were significant differences in FMA-UE, ARAT, and BBT scores (all *P*<.001) between the experimental and control groups. The MCID findings illustrated that the experimental group had clinically meaningful improvements compared to the control group. There were significant correlations between electromyography signal features and clinical outcome measures (all *P*<.05) in both groups after rehabilitation. However, the experimental group exhibited strong positive correlations, while the control group exhibited moderate positive correlations. At follow-up, the mean movement accuracy was notably higher in the experimental group than in the control group (mean 83.59%, SD 1.1% vs mean 79.20%, SD 0.8%), indicating that hand motor function was effectively sustained through the use of the VR-based intervention in the experimental group.

**Conclusions:**

The findings of this study revealed that VR-based hand games with enhanced visual training feedback substantially improved hand motor function in patients with subacute stroke.

## Introduction

### Background

A stroke results in physical impairments that restrict a person’s ability to engage in everyday activities. Stroke survivors need their upper extremities to be more controllable and adaptive for improving their motor recovery [1,2]. Conventional physical therapy for patients with stroke has evolved into advanced rehabilitation because of technological advancements [3]. The brain, through motor learning, adjusts to new situations, which results in neuronal plasticity and the creation of new neural networks [4]. To improve plasticity, consideration is given to movements that are task-oriented and recurring [5,6].

Virtual reality (VR) studies have implied that VR-based therapy improves neuroplasticity through motor movements in a VR environment [7]. The primary improvement in motor performance has been attributed to the strengthening of neural pathways, which can be achieved through visual feedback training that includes task-specific and repetitive workouts based on VR games [8]. Because of the interactive environment and the visual feedback, patients with stroke continue performing task-oriented VR game–based exercises that allow them to track their progress, making it feasible to achieve functional goals. Recent scientific developments have led to the creation of fully immersive VR devices, which can overcome the limitations of semi-immersive VR technologies [9].

Although traditional physical therapy and robotics are applied in stroke rehabilitation, they often cause limitations in achieving long-term improvements. These methods emphasize task-specific and physical exercises, which become tedious, reduce therapy motivation, and reduce patient involvement. Another main obstacle to effective stroke recovery is the repetitiveness of traditional exercises without any interactive engagement and motivation. This leads to decreasing repetitions of exercise that can ultimately limit the effectiveness of rehabilitation. Consequently, this results in slow progress in improving hand motor function, leading to inadequate compliance with therapy.

Furthermore, it has been revealed that VR-based rehabilitation increases the motivation and enjoyment of patients with stroke during therapy [9]. However, these studies concentrated mainly on motivation for improvement and neglected to sustain long-lasting improvements in hand motor function. Therefore, sustaining enduring improvements in motor function necessitates the potential integration of cognitive engagement within visual feedback. Implementing such approaches can expedite motor learning, substantially enhancing hand motor function.

### Literature Review

Electromyography (EMG) has been extensively used in clinical research involving neurorehabilitation as an objective measure for functional status assessment and muscle strength [10]. Ying et al [5] created an exoskeleton arm for stroke survivors, and surface EMG signals were used for repeated training to examine distinctions in muscle amplitude. Classifier performance has been improved and developed for different features of EMG. Feature extraction techniques allow EMG signals to characterize and interpret the information for intentions and motor tasks associated with stroke rehabilitation [11]. Zhang and Zhou [12] suggested an investigative approach for intention potential in pattern recognition–based rehabilitation, using the performance of classification and high-density surface EMG.

When comparing fully immersive VR systems to semi-immersive VR systems, it can be observed that the latter systems are much more advanced and provide 3D graphics [3,13,14]. Rodríguez-Hernández et al [15] used Rheometric software to assess the combined effects of traditional therapy and VR on the motor functions of the upper extremities in patients with stroke. Moreover, Shahmoradi et al [16] used VR games in chronic stroke survivors.

According to Brannstrom recovery phases, findings indicate that playing games can improve participants’ range of motion (ROM) [17]. A significant role in the process of adaptation to movements is played by the enhancement of neuroplasticity that occurs because of the successful use of visual feedback [17].

Subjective assessments in clinical settings and scenarios mostly fall into several scales based on clinical experience measurement techniques [3]. These include the Barthel Index [18], Action Research Arm Test (ARAT) [19], Box and Block Test (BBT) [20], and Fugl-Meyer Assessment-upper extremity (FMA-UE) [1]. The minimal clinically meaningful difference (MCID) signifies the minimal change in an outcome measure considered meaningful in clinical and research practice [21]. The MCID ascertains whether intervention score changes reflect a clinically meaningful patient improvement [22]. In subjective assessment, a doctor’s training and expertise determine the reliability of the results of the clinical outcome measure examination [23]. Stroke survivors’ physiological data [24], such as EMG signals, are used to conduct a functional assessment of the movements of patients with stroke [25].

### Study Aim

This research aimed to evaluate the effectiveness of VR-based games for achieving enduring improvements in hand motor function, using EMG signals. This longitudinal assessment provides insights into the correlation among EMG signal features, clinical outcome measures (FMA-UE, ARAT, and BBT), and weekly progression in movement performance. The following VR-based hand games were developed: hit a rolling ball, grasp a balloon, swap hands, and grip a pencil. The movements in the VR-based hand games included flexion and extension, opening and closing, supination and pronation, and pinching. Correlations were evaluated before and after the intervention and at follow-up. Movement classification was investigated for movement performance progression at baseline, week 4, week 6, and week 9 (follow-up) using k-nearest neighbor (KNN), random forest (RF), and support vector machine (SVM) classifiers.

This research marks a valuable contribution to the EMG-based assessment of the effectiveness of customized, fully immersive VR-based hand games. These games used an innovative approach of integrating cognitive engagement within visual training feedback to attain long-lasting improvements.

## Methods

### Implementation of Rehabilitation Games Using Unity3D and a VR Headset

**Figure 1.**
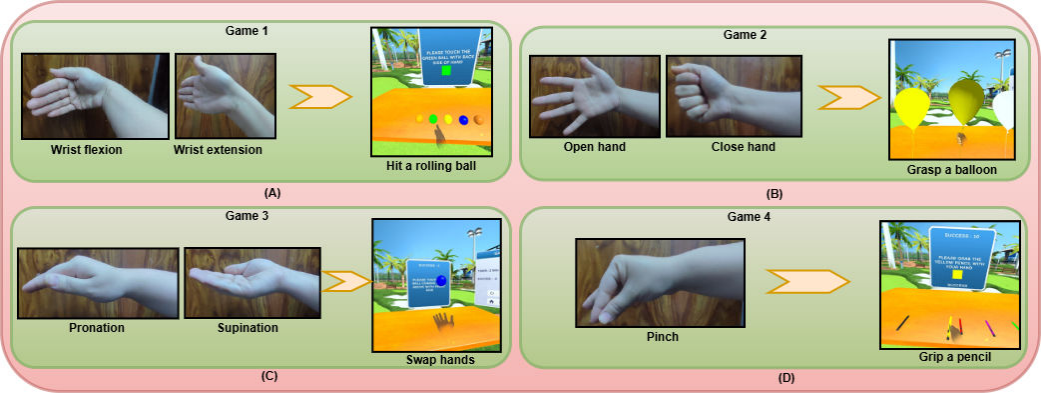
Virtual reality–based rehabilitation games based on hand movements: (A) wrist flexion and extension, (B) hand opening and closing, (C) pronation and supination, and (D) pinch.

VR games were created for individuals with subacute stroke to help with hand rehabilitation. Four VR games were designed around hand movements, specifically flexion and extension (game 1: hit a rolling ball), opening and closing (game 2: grasp a balloon), supination and pronation (game 3: swap hands), and pinching (game 4: grip a pencil) (Figure 1). Visuals of the VR games are provided in Multimedia Appendix 1. The Unity3D game engine (version 2021.2.12f1; long-term support release) was used to develop the rehabilitation games. The Oculus Quest 2 VR device (processor: Qualcomm Snapdragon XR2; RAM: 6 GB; display: 1832×1920 pixels per eye; refresh rate: supports up to 120 Hz; field of view: approximately 100°; tracking: inside-out, 6 degrees of freedom, with hand-tracking capability via integrated cameras and controller input; operating system: Android-based Oculus OS; wireless connectivity: supports Wi-Fi 6 [802.11ax]) was used to run the games. Regarding system resource usage during gameplay, GPU utilization averaged 72%‐80% and CPU utilization averaged 60%‐70%. Moreover, the headset maintained a stable 72‐90 frames per second without any visual discomfort or motion sickness, and memory utilization stayed within safe levels (less than 75% of the available RAM).

Unity3D was selected for its reliability, enduring upgrades, high-quality physics and rendering, and robust compatibility with the Oculus Quest 2 VR device. Unity3D features native VR integration with the Oculus software development kit (SDK), ensuring dependable hand tracking and minimal latency, which are crucial for real-time rehabilitation activities. Moreover, Unity’s C# environment made it possible to efficiently implement custom interaction logic and adaptive game mechanics. In addition, Unity offers a free license for academic and research purposes. Regarding runtime performance, no runtime crashes, overheating, or motion-latency issues were observed, which could adversely impact patient experience. Minor frame drops occurred when rendering multiple complex objects simultaneously; however, these were infrequent and did not interfere with the operations of the rehabilitation tasks.

Patients with stroke experienced cognitive engagement paradigms within visual feedback that involved attention, with patients selecting an object’s color while focusing on visual cues, and decision-making engagement involved correctly choosing the color of an object following the appearance of objects randomly with varying colors on a table. In the games, patients used hand-tracking technology within the VR headset and observed their affected hand moving and interacting with virtual objects.

### Game Development Process

Game development was carried out using Unity3D (2021.2.12f1) integrated with the Oculus SDK, which targeted the Oculus Quest 2 platform. Unity’s XR Hand Tracking API was used to provide naturalistic hand interactions, which were enabled by implementing an interaction model based on inside-out tracking with hand tracking. Custom C# control scripts were used to map kinematic inputs, such as hand flexion, pinch detection, and rotation angles, to interaction states, such as grasp, release, and hit. The built-in PhysX engine was used to simulate object dynamics, such as gravity, collision, and resistance. To minimize clutter and maintain immersion, the user interface was created with only a few world-space overlays, such as progress bars and score counters. To enhance user involvement, instant feedback mechanisms were incorporated, which combined audio signals, such as success tones, to reward proper motions with visual cues, including object color changes and animations. To minimize GPU load and preserve shape recognition, optimization was given priority while rendering objects with a low polygon count. This ensured fluid performance at a consistent frame rate of 72 frames per second in all games. Ultimately, the project was built and deployed as an Android package kit (APK) to run on the stand-alone VR headset Oculus Quest 2.

### Immersive VR Game Design Mechanics

The developed VR-based hand games for hand rehabilitation are presented in Figure 1. Descriptions of the VR games are provided below.

#### Game 1: Hit a Rolling Ball

The patient approaches randomly generated rolling balls of various colors on a virtual table and flexes the affected wrist to strike a specific ball with the palm of their hand. Subsequently, the patient extends the same wrist to strike a specific ball with the back of their hand. This game challenges the patient to have exact control over wrist flexion and extension to time the strike properly. Faster speeds at more challenging levels require enhanced motor coordination, reaction time, and sustained attention.

#### Game 2: Grasp a Balloon

The patient approaches randomly generated balloons of various colors on a virtual table. The patient closes the affected hand to pick up a specific balloon (grasping movement). Once the hand is closed, the patient holds the balloon for 5 seconds. This holding of the balloon helps to strengthen the hand muscles. After the grasping time, the balloon is released, and the hand is considered to be open and ready for the subsequent interaction. This game challenges the patient to sustain grasping without exerting excessive effort and to pay selective attention to the target. It emphasizes grasping precision and decision-making.

#### Game 3: Swap Hands

The patient extends the affected hand in a supinated position, and a randomly generated virtual ball descends from the top with appropriate speed and touches the palm of the patient’s hand. The patient then instantly swaps their hand from the supinated position to a pronated position, and another virtual ball descends and touches the back of the patient’s hand. This game challenges the patient to rotate their forearm quickly, precisely, and with coordination. At higher levels, the quick transition demand makes it more challenging to sustain controlled and smooth movements. The game emphasizes movement adaptability and responsiveness.

#### Game 4: Grip a Pencil

The patient approaches randomly generated pencils of various colors with wide gaps on a virtual table. The patient pinches a specific pencil with the affected hand to pick it up. The pencil is held for 5 seconds before placing it back on the virtual table. This game challenges the patient to develop stability when gripping a small object, fine motor skills, and selective color recognition. Narrow spacing between pencils requires more dexterity and increases the risk of errors. The game emphasizes pinch grip strength and accuracy.

### Ethical Considerations

**Figure 2.**
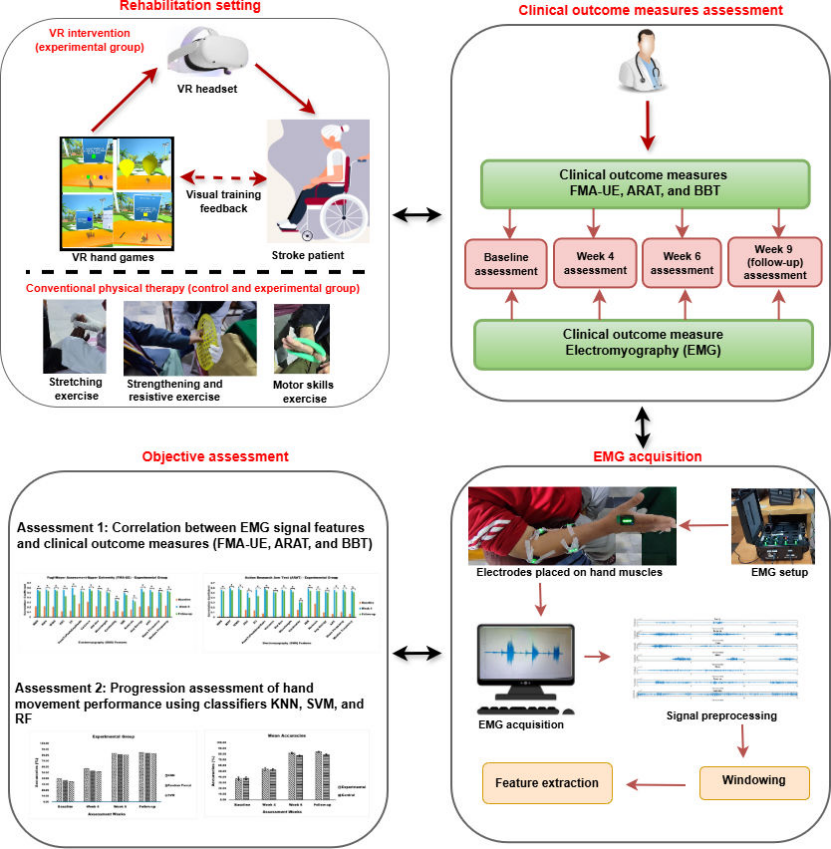
The rehabilitation settings and assessment framework used in this research. ARAT: Action Research Arm Test; BBT: Box and Block Test; FMA-UE: Fugl-Meyer Assessment-upper extremity; KNN: k-nearest neighbor; RF: random forest; SVM: support vector machine; VR: virtual reality.

This study was carried out in the physiotherapy department of a hospital. The study was approved by the research ethics committee (reference number: BMES/REC/22/027), and it followed the institutional guidelines and the principles of the Declaration of Helsinki. The study was registered on ClinicalTrials.gov (NCT06582303). Registration was completed retrospectively with respect to participant enrollment but prospectively with respect to data analysis and outcome assessment. Written informed consent was obtained from each patient for participation in the study. No compensation was provided to the participants. All collected data have been treated anonymously, and identifiable information has not been linked to research data. Figure 2 illustrates the rehabilitation setting and assessment framework used in this research.

### Inclusion and Exclusion Criteria

Based on the inclusion and exclusion criteria, patients were registered in this study. The inclusion criteria were as follows: (1) Modified Ashworth Scale score <4, (2) Fugl-Meyer Assessment-upper limb score between 25 and 55, (3) Montreal Cognitive Assessment score ≥21, (4) subacute stroke type according to the American Stroke Association (ASA) and existing literature (the subacute phase of stroke is generally described as the period ranging from 2 weeks to 6 months after stroke onset), and (5) age ≥18 years.

The exclusion criteria were as follows: (1) contractures resulting from burns, (2) wrist impairments caused by muscular disorders, (3) presence of external fixation devices, and (4) presence of vestibular problems.

### Sample Size and Recruitment

G*Power software 3.9.1.4 (Heinrich-Heine-Universität Düsseldorf) was used to evaluate the sample size for this study. The parameters included an effect size (d) of 0.80, α error of .05, and power of 0.80. Considering a dropout rate of 10%, 56 individuals with subacute stroke were included. Four patients who did not meet the eligibility criteria were excluded from the trial.

Patients were recruited from the physiotherapy department of the hospital. During the initial screening, patients with a diagnosis of subacute stroke were first identified by a physiotherapist. Clinical coordinators who were trained physiotherapists on our team then directly approached these patients in the outpatient waiting areas. The coordinators explained the study’s purpose, procedures, potential risks, and benefits. Patients who expressed interest in participating underwent screening against the predefined inclusion and exclusion criteria. Those who met all the eligibility criteria were invited to participate. Written informed consent was obtained before enrollment, and participants underwent randomization.

This study used the sealed envelope method to ensure proper allocation concealment. The randomization procedure was conducted by an independent individual who was not involved in any aspect of the study. This person used the sealed envelope method to allocate participants equally into the experimental and control groups. Before the trial began, a randomization sequence was created to determine the assignment of participants to either the experimental or control group. Each assignment was placed inside an identical, sequentially numbered, opaque, and sealed envelope to prevent prior knowledge of the allocation. Patients were assigned the next available sequentially numbered envelope when they were confirmed eligible and provided informed consent. The envelope was only opened after recording participants’ details, ensuring the allocation process remained concealed.

### Interventions for the Experimental and Control Groups

**Figure 3.**
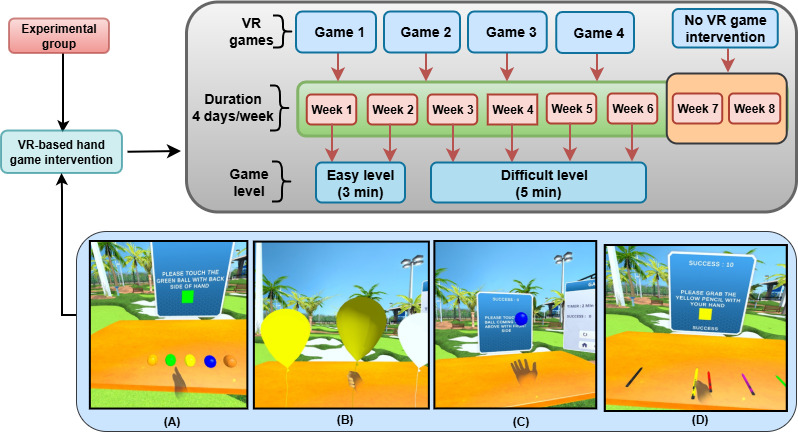
Virtual reality (VR) game intervention protocol in the experimental group. The games are as follows: (A) hit a rolling ball, (B) grasp a balloon, (C) swap hands, and (D) grip a pencil [26].

The experimental group received both VR game therapy and conventional physical therapy, while the control group only received conventional physical therapy. The experimental group used the VR game intervention protocol paradigm for hand rehabilitation games, as shown in Figure 3. Conventional physical therapy included ROM exercises, stretching, resistance exercises, and strengthening exercises. Both groups participated in intervention sessions 4 days a week for 6 weeks (24 sessions in total). Moreover, both groups completed 2 weeks of follow-up. The experimental group received 24 minutes of VR game therapy and 24 minutes of conventional physical therapy per session in weeks 1 and 2, and 40 minutes of VR game therapy and 40 minutes of conventional physical therapy per session in weeks 3-6. The control group received 48 minutes of conventional physical therapy per session in weeks 1 and 2, and 80 minutes of conventional physical therapy per session in weeks 3-6. The conventional physical therapy protocol included ROM exercises for the joints (shoulder, elbow, and wrist), strengthening and resistance exercises for weak muscles (using a power web and gym equipment), muscle stretching (shoulder flexors, abductors, external rotators, elbow and wrist extensors, and hand musculature), and motor skills training for the upper limb (Thera putty and occupational therapy tools). The amount of time spent on VR game therapy and conventional therapy in the experimental group was equivalent to the amount of time spent on conventional therapy in the control group.

After week 2, the extended intervention duration in the experimental group allowed patients to increase their repetition volume in more challenging conditions, which in turn improved their intensity and functional engagement. Similarly, the longer duration in the control group allowed for increased repetition of conventional exercises at higher volume. Consequently, to ensure that any disparities in outcomes were not the result of unequal therapy exposure but rather the result of the qualitative content of the intervention, all groups were subjected to the same escalation in therapy time.

Due to the nature of the intervention, which necessitated active participation and interaction with the VR system, blinding participants and therapists was not feasible. To minimize potential bias, we standardized training techniques and provided comprehensive guidelines to physiotherapists to ensure uniformity in implementation across groups. Patients were also provided with uniform instructions to ensure consistency in engagement across participants.

### Outcome Measures

#### Overview

The primary outcome measures included the correlation between EMG signal features and clinical outcome measures (FMA-UE, ARAT, and BBT), and the weekly progression of movement performance. FMA-UE, ARAT, and BBT outcome measures have been evaluated and discussed in [26] for the experimental group and the control group. These were used to evaluate the efficacy of the hand’s motor function. The MCID values of the FMA-UE, ARAT, and BBT reported in previous studies [21,27,28] serve as benchmarks for interpreting the progression of the clinical outcome measures of subacute stroke. The clinical outcome measures (FMA-UE, ARAT, and BBT) are explained below.

#### FMA-UE Outcome Measure

The FMA-UE was used to assess stroke survivors’ upper extremities for the recovery of motor function, which involved performance-based measurement. The FMA-UE had high inter- and intrarater reliability (*r*=0.98‐0.99), responsiveness, and construct validity in previous studies [21,22]. FMA-UE construct validity has shown significant correlations with the Functional Independence Measure and the ARAT, with reported correlation values of 0.61‐0.94 [22]. This scale includes 33 items used to evaluate motor function. The grading criteria range from 0 to 66, with a score of 0 denoting severe impairment and 66 denoting normal motor function. A higher score is indicative of an improvement in motor function for the individual. The FMA-UE was administered by a physical therapist in a clinical setting. The reported MCID of the FMA-UE for the subacute stroke stage is 9 points [21,29].

#### ARAT Outcome Measure

The ARAT evaluates the functional ability performance of the upper limbs of stroke survivors. This test emphasizes reaching, grasping, pinching, and general movement activities. ARAT performance was found to be significantly correlated with FMA-UE and BBT performance. Prior studies revealed high intra- and interrater reliability (intercorrelation coefficient >0.98) and validity (*r*=0.81‐0.97) of the ARAT to evaluate the functional ability performance of stroke survivors [30,31]. The overall score ranges from 0 to 57, with each item rated on a 4-point scale. Severe disability is indicated by the lowest score of 0, while normal upper extremity function is determined by the highest score of 57 [30]. A higher score on the ARAT is indicative of enhanced functional capabilities of the upper extremities. A physical therapist administered the ARAT in a clinical setting. The reported MCID of the ARAT for stroke is 6 points [27,32].

#### BBT Outcome Measure

The BBT is used to evaluate the hand dexterity of patients who have experienced a stroke. The BBT has been identified as a reliable and valid assessment method for hand dexterity, including fine motor skills, in prior investigations [33]. Patients are instructed to transfer wooden blocks from one compartment to another within a time limit of 1 minute. The final score is determined by the number of wooden blocks transferred during the 1-minute trial [27]. The BBT was administered by a physical therapist in a clinical setting. The reported MCID of the BBT for stroke is 6 points [27,28,32].

#### EMG Assessment

##### EMG Acquisition

**Figure 4.**
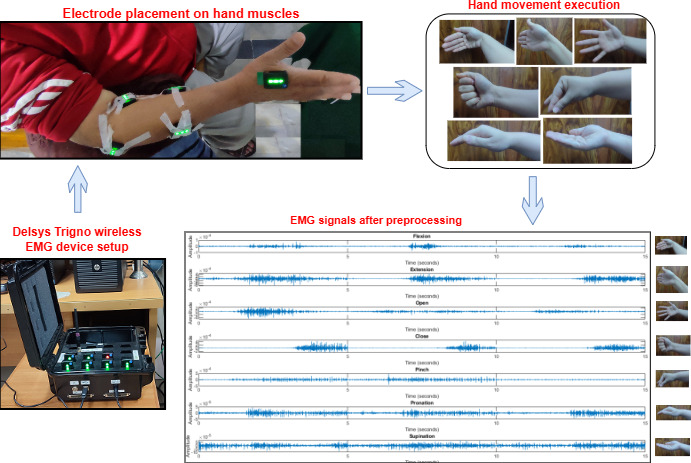
Electromyography (EMG) acquisition. EMG signals are recorded using the Delsys Trigno wireless EMG system. EMG electrodes are placed on 5 forearm muscles, including the extensor digitorum communis, flexor digitorum superficialis, supinator, pronator teres, and dorsal interossei. The EMG signals show hand movements that include flexion, extension, opening, closing, pinch, pronation, and supination.

EMG signals were recorded for 7 movements by using the Delsys Trigno Wireless EMG system, which has a sampling frequency of 1926 Hz. EMG electrodes were placed on 5 forearm muscles, including the extensor digitorum communis, flexor digitorum superficialis, supinator, pronator teres, and dorsal interossei. The hand movements included flexion, extension, opening, closing, pinch, pronation, and supination. EMG signals were recorded at baseline, week 4, week 6, and week 9 (follow-up). Each patient was positioned upright on a chair in front of an LCD screen. A MATLAB graphical user interface was developed to assist patients in performing the required movements with the help of visual cues. Every patient in the EMG recording session executed the protocol by initially keeping their affected hand at rest for 5 seconds and then using their muscles to contract for a further 5 seconds to complete the movement. Each movement was performed 3 times with relaxation intervals between the repetitions. Each movement and relaxation repetition lasted for 5 seconds. EMG acquisition is presented in Figure 4.

##### EMG Preprocessing

EMG signals are intrinsically contaminated with noise due to power line interference and motion artifacts. Therefore, it is of utmost importance to remove the noise. Power line noise was removed by using a 50-Hz notch filter. A fourth-order Butterworth bandpass filter of 20‐450 Hz was applied to remove motion artifacts and noise from the acquired EMG signal data. In the next step, EMG signals corresponding to movements were separated from the rest of the EMG signals to ensure increased prediction accuracy and boost training. The windowing technique ensures a continuous distribution of pertinent data [34,35]. A window in the overlap windowing approach is described by both the window size and the step size, as opposed to the disjoint windowing technique, where a window is characterized by its size. Following preprocessing, this assessment used overlapping windowing segmentation with a window size of 250 ms and an overlapping size of 50% (125 ms). Since it contains the most significant information in the signal, most researchers have included it in their studies [36].

##### EMG Feature Extraction

The EMG signal features used in this study are based on both time-domain features and frequency-domain features. The time-domain features include root mean square, mean absolute value, integrated EMG, slope sign change, zero crossing, peak-to-peak amplitude, variance, SD, waveform length, cardinality, IQR, kurtosis, average energy, and average amplitude change. The frequency-domain features include mean frequency and median frequency. Both feature groups are being widely used in EMG research and practice [35,37-41]. These features were extracted from each window segment obtained in the previous step.

### Statistical Analysis of Correlations and Movement Performance

Data from the experimental and control groups were analyzed using SPSS 21 statistical software (IBM Corp). Variables were tested for normality using the Shapiro-Wilk test. The correlation between EMG signal features and clinical outcome measures was examined using the Spearman rank correlation coefficient. The statistical significance was set at *P*<.05. Machine learning techniques were applied to the EMG data of stroke patients to evaluate the movement performance of the experimental and control groups at assessment points, including baseline, week 4, week 6, and week 9 (follow-up). Three classifiers (KNN, RF, and SVM) were used for movement performance. These classifiers have been extensively used on EMG data for different movement classifications and muscle activities [39,42-44] and have therefore been adopted in this study. KNN assigns class labels according to the Euclidean distance to the closest neighbors in the feature space. RF constructs ensembles of decision trees by partitioning the feature space into several segments. SVM identifies an ideal hyperplane, whether linear or kernel-based, to distinguish between classes. The classifiers were tested for normality using the Shapiro-Wilk test. Movement performance was tested using ANOVA and the paired 2-tailed *t* test.

## Results

### Analysis Structure

**Figure 5.**
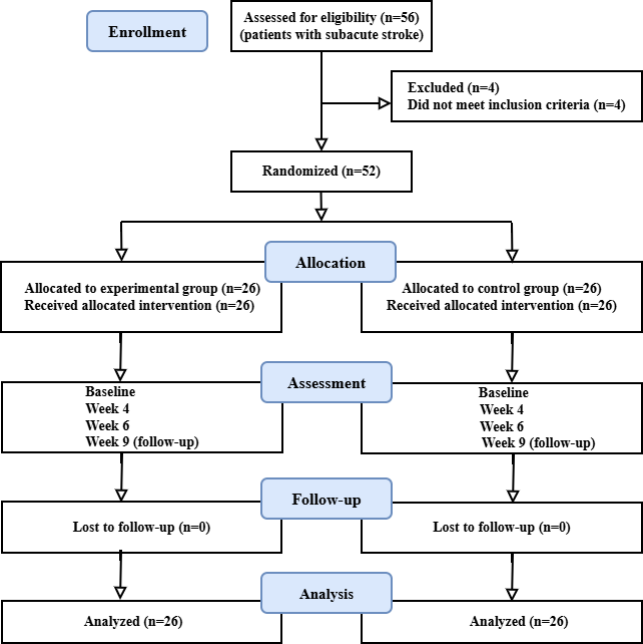
CONSORT (Consolidated Standards of Reporting Trials) flowchart.

The participants in this research were 52 patients with subacute stroke who met the eligibility criteria. These patients were randomly allocated to the experimental and control groups, with 26 patients in each group. The results have been divided into three parts: (1) comparison of clinical outcome measure scores with the MCID, (2) examination of the correlation between muscle activity (EMG) and hand function clinical outcome measures, and (3) assessment of the weekly progress of hand movement performance using EMG-based classification accuracies. Figure 5 presents the CONSORT (Consolidated Standards of Reporting Trials) [45] flow diagram. The CONSORT-EHEALTH checklist is provided in Checklist 1.

### Demographic and Clinical Data

Demographic and clinical data are presented in Table 1. The Mann-Whitney *U* test revealed no significant differences in characteristics between the experimental and control groups (all *P*>.05). Furthermore, the *P* values for demographic and clinical data were high, indicating that the groups exhibited comparable variations.

**Table 1.** Demographic and clinical data.

Characteristic	Control group (n=26)	Experimental group (n=26)	*P* value^a^
Gender, n (%)			.56
Male	18 (69)	16 (62)	
Female	8 (31)	10 (38)	
Age (years), mean (SD)	49.8 (9.9)	51.8 (12.9)	.78
Affected hand, n (%)			.27
Right	15 (58)	11 (42)	
Left	11 (42)	15 (58)	
MoCA^b^, mean (SD)	22.5 (1.9)	22.6 (1.9)	.58
FMA-UE^c^, mean (SD)	36.6 (7.4)	40.84 (8.6)	.19
MAS^d^, n (%)			.32
Grade 0	7 (27)	10 (39)	
Grade 1	7 (27)	7 (27)	
Grade 1+	6 (23)	5 (19)	
Grade 2	6 (23)	4 (15)	

a Mann-Whitney *U* test.

b MoCA: Montreal Cognitive Assessment.

c FMA-UE: Fugl-Meyer Assessment-upper extremity.

d MAS: Modified Ashworth Scale.

### Comparison of Clinical Outcome Measure Scores With the MCID

The mean differences in the clinical outcome measure instruments (FMA-EU, ARAT, and BBT) between groups and their comparisons with the MCID are presented in Table 2 [26]. At baseline (preintervention), there were no significant differences in the scores of FMA-UE (*P*=.19), ARAT (*P*=.13), or BBT (*P*=.09) between the experimental and control groups. However, at follow-up (postintervention), there were significant differences in the scores of all outcome measures (all *P*<.001) between the experimental and control groups. All mean differences in FMA-UE, ARAT, and BBT scores were greater than the corresponding MCIDs (9, 6, and 6 points, respectively).

**Table 2.** Clinical outcome measure scores and comparison with the MCID^a^.

Clinical outcome measure and assessment time point	Experimental group, mean (SD)	Control group, mean (SD)	*P* value^b^	Mean difference between groups	Comparison with the MCID
FMA-UE^c^ score				9.46	Greater than the MCID of 9 points
Preintervention (baseline)	40.84 (8.6)	36.61 (7.4)	.19		
Postintervention (follow-up)	55.92 (7.3)	42.23 (7.9)	<.001		
ARAT^d^ score				8.96	Greater than the MCID of 6 points
Preintervention (baseline)	24.23 (4.9)	21.23 (8.2)	.13		
Postintervention (follow-up)	37.46 (9.1)	25.50 (6.7)	<.001		
BBT^e^ score				20.46	Greater than the MCID of 6 points
Preintervention (baseline)	15.11 (6.7)	11.76 (8.6)	.09		
Postintervention (follow-up)	39.50 (12.8)	15.69 (8.4)	<.001		

a MCID: minimal clinically meaningful difference.

b Mann-Whitney *U* test for comparison between the experimental and control groups.

c FMA-UE: Fugl-Meyer Assessment-upper extremity.

d ARAT: Action Research Arm Test.

e BBT: Box and Block Test.

### Correlations Between EMG Signal Features and Clinical Outcome Measures

**Figure 6.**
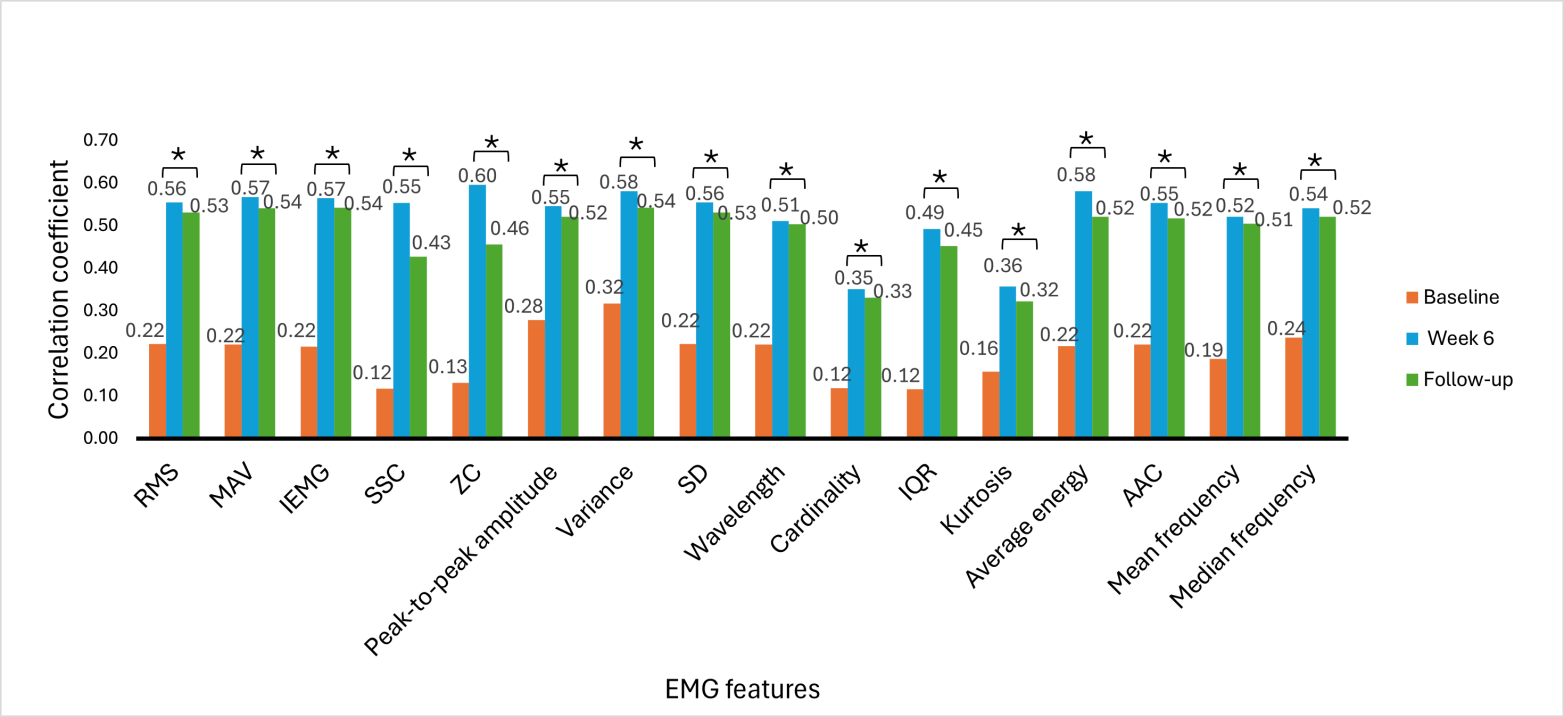
Correlation between electromyography (EMG) signal features and Fugl-Meyer Assessment-upper extremity scores at each time measurement in the experimental group. AAC: average amplitude change; IEMG: integrated EMG; MAV: mean absolute value; RMS: root mean square; SSC: slope sign change; ZC: zero crossing. *P<.05.

**Figure 7.**
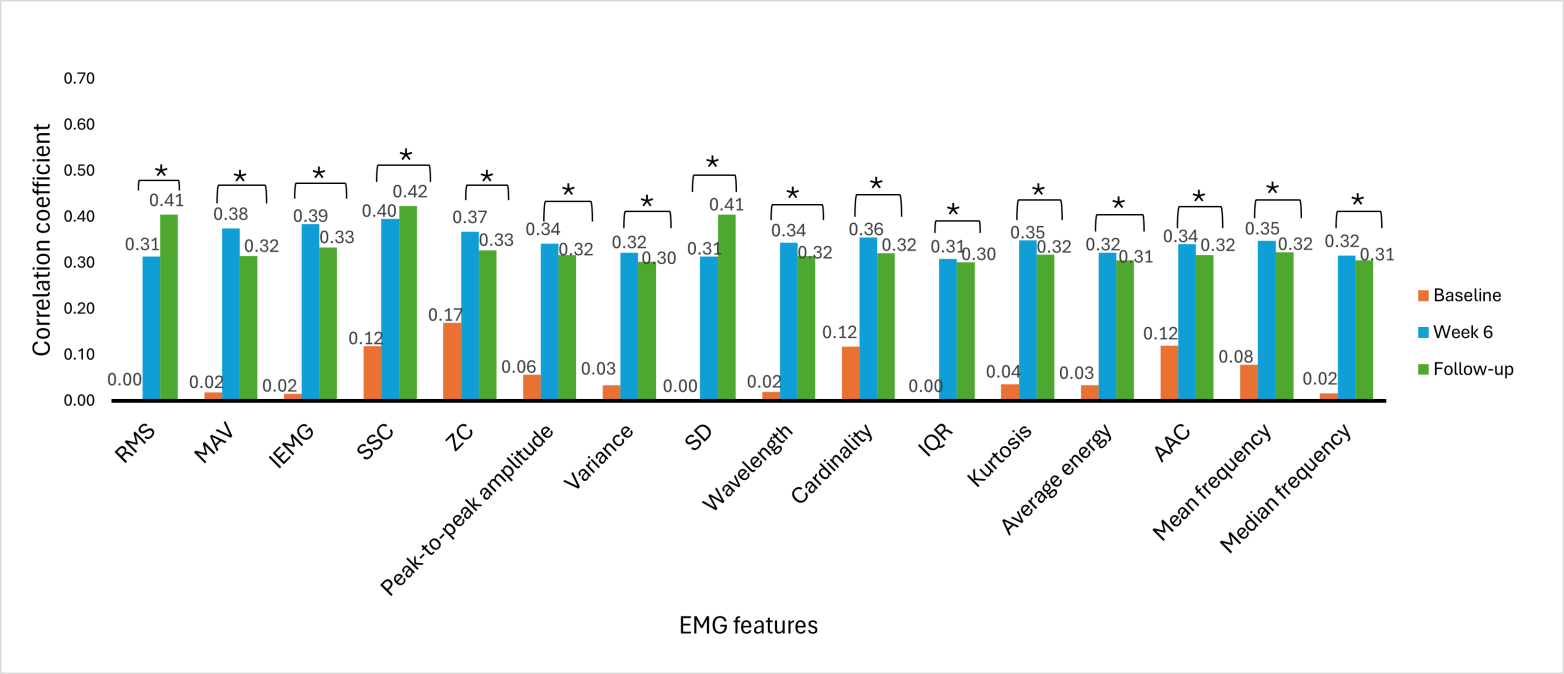
Correlation between electromyography (EMG) signal features and Fugl-Meyer Assessment-upper extremity scores at each time measurement in the control group. AAC: average amplitude change; IEMG: integrated EMG; MAV: mean absolute value; RMS: root mean square; SSC: slope sign change; ZC: zero crossing. *P<.05.

**Figure 8.**
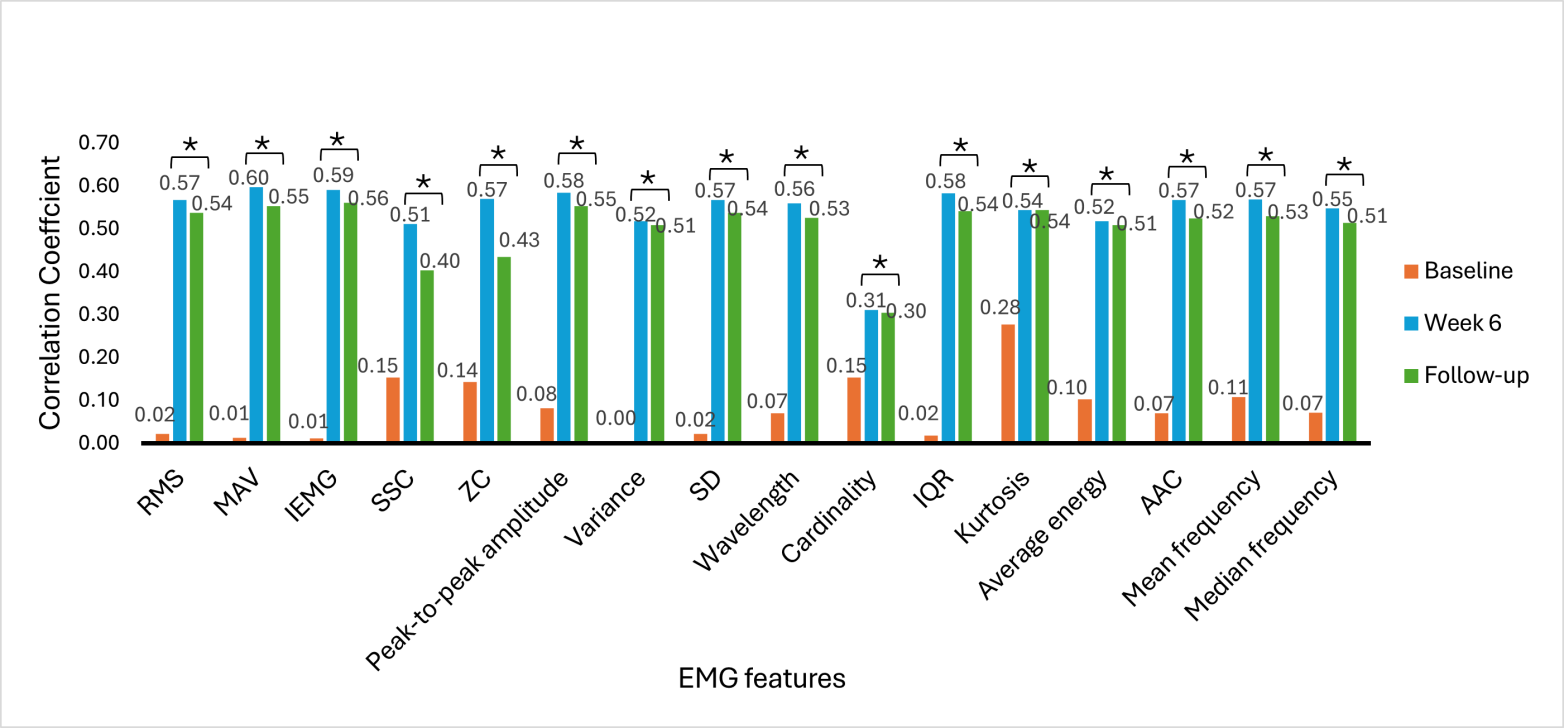
Correlation between electromyography (EMG) signal features and Action Research Arm Test scores at each time measurement in the experimental group. AAC: average amplitude change; IEMG: integrated EMG; MAV: mean absolute value; RMS: root mean square; SSC: slope sign change; ZC: zero crossing. *P<.05.

**Figure 9.**
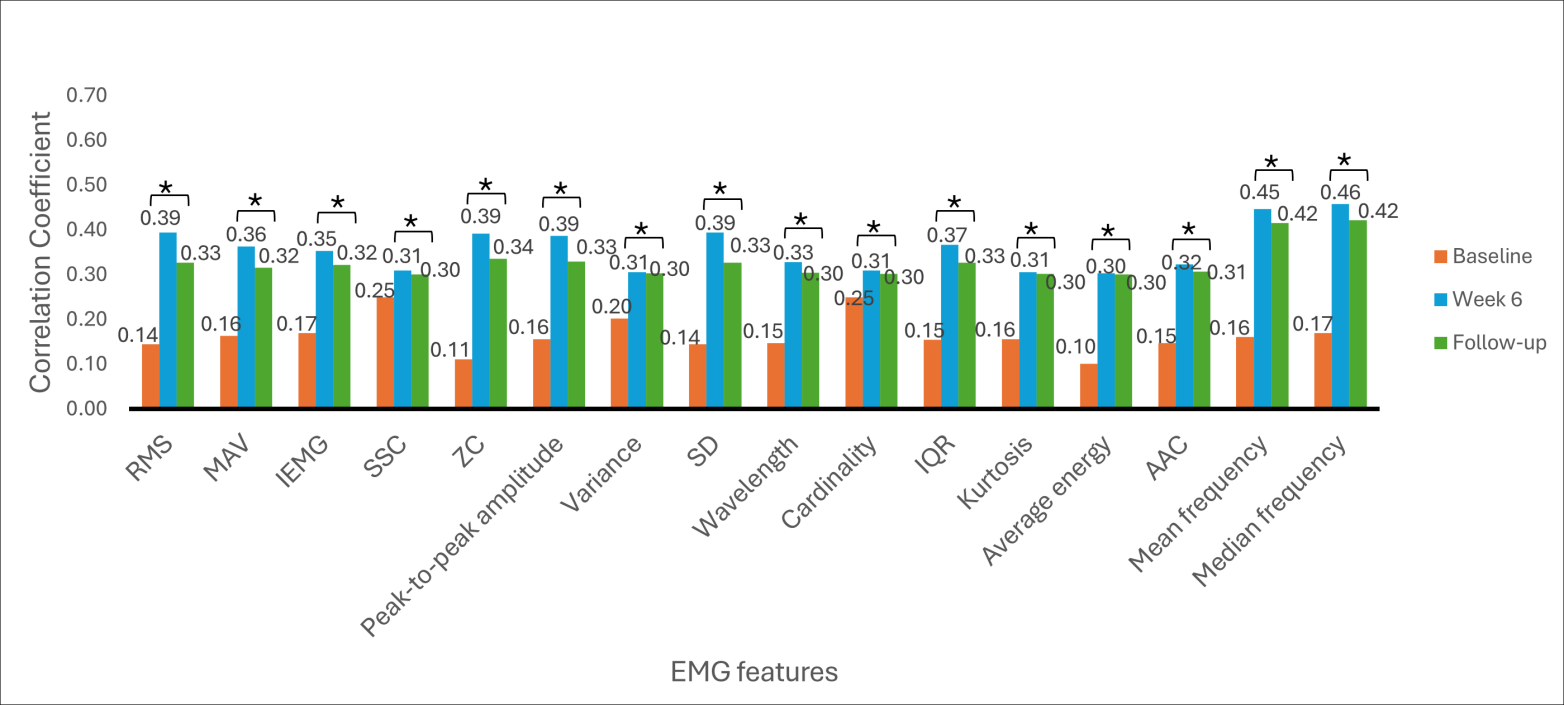
Correlation between electromyography (EMG) signal features and Action Research Arm Test scores at each time measurement in the control group. AAC: average amplitude change; IEMG: integrated EMG; MAV: mean absolute value; RMS: root mean square; SSC: slope sign change; ZC: zero crossing. *P<.05.

**Figure 10.**
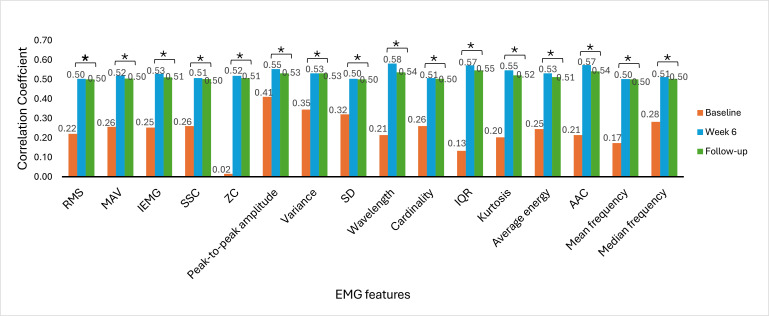
Correlation between electromyography (EMG) signal features and Box and Block Test scores at each time measurement in the experimental group. AAC: average amplitude change; IEMG: integrated EMG; MAV: mean absolute value; RMS: root mean square; SSC: slope sign change; ZC: zero crossing. *P<.05.

**Figure 11.**
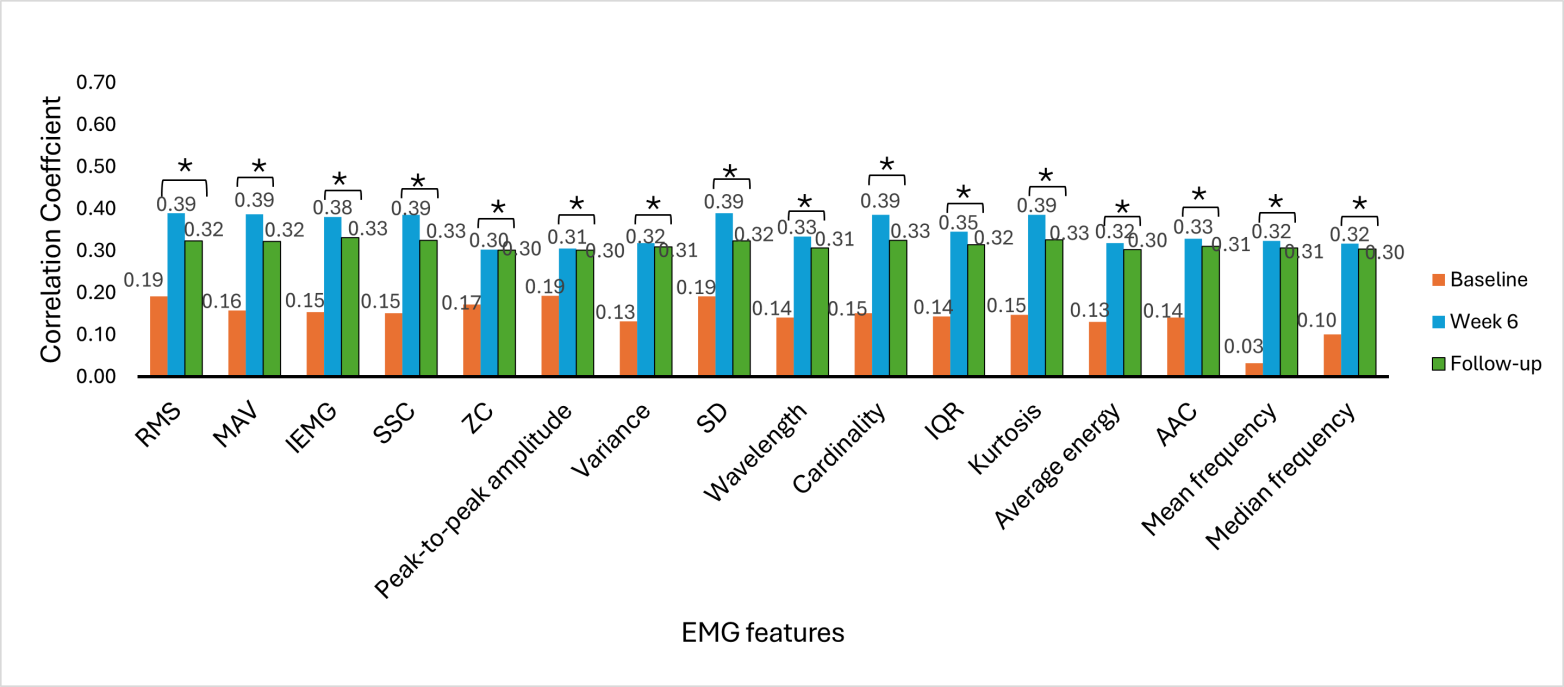
Correlation between electromyography (EMG) signal features and Box and Block Test scores at each time measurement in the control group. AAC: average amplitude change; IEMG: integrated EMG; MAV: mean absolute value; RMS: root mean square; SSC: slope sign change; ZC: zero crossing. *P<.05.

The Spearman rank correlation coefficient was used to determine the correlation between EMG signal features and clinical outcome measures. The correlation between EMG signal features and clinical outcome measures provides insights into the association of muscle activity with motor function recovery, functional abilities, and hand dexterity variability among stroke survivors. The correlation was analyzed at baseline, week 6, and week 9 (follow-up). Figures 6-11 illustrate the results of the correlations between the experimental and control groups. Both groups exhibited a weak and nonsignificant (all *P*>.05) relationship between EMG features and clinical outcome measures at baseline. Results at follow-up revealed significant (all *P*<.05) strong positive correlations in the experimental group and significant (all *P*<.05) moderate positive correlations in the control group.

### Progression Analysis of Movement Performance

Machine learning algorithms were used on the EMG data of patients with stroke to assess the progression of movement performance at different time points in the experimental and control groups. The analysis focused on the weekly movement performance measured by the classification accuracy of EMG signals. There were significant differences between the paired weeks in KNN, RF, and SVM values (all *P*<.05) (Table 3). Furthermore, there were significant differences between the 2 groups in KNN, RF, and SVM values (all *P*<.001) (Table 3).

**Table 3.** Effect of virtual reality games on the progression of weekly movement performance.

Classifier	Assessment time points				*P* value^a^ for difference between groups
	Baseline and week 4	Week 4 and week 6	Week 6 and week 9	Week 9 (follow-up) and baseline	
KNN^b^					<.001
Value, mean (SD)	15.93 (1.6)	24.61 (2.1)	1.59 (0.1)	42.14 (3.6)	
*P* value^c^	.045	.04	.04	.04	
RF^d^					<.001
Value, mean (SD)	15.26 (1.2)	27.34 (1.6)	1.94 (0.1)	44.50 (2.9)	
*P* value^c^	.04	.03	.002	.03	
SVM^e^					<.001
Value, mean (SD)	16.16 (1.3)	27.20 (1.7)	1.99 (0.1)	45.37 (3.2)	
*P* value^c^	.04	.03	.02	.03	

a ANOVA for mean difference comparison between the experimental and control groups.

b KNN: k-nearest neighbor.

c Paired *t* test for mean difference comparison between time points within the experimental and control groups.

d RF: random forest.

e SVM: support vector machine.

**Figure 12.**
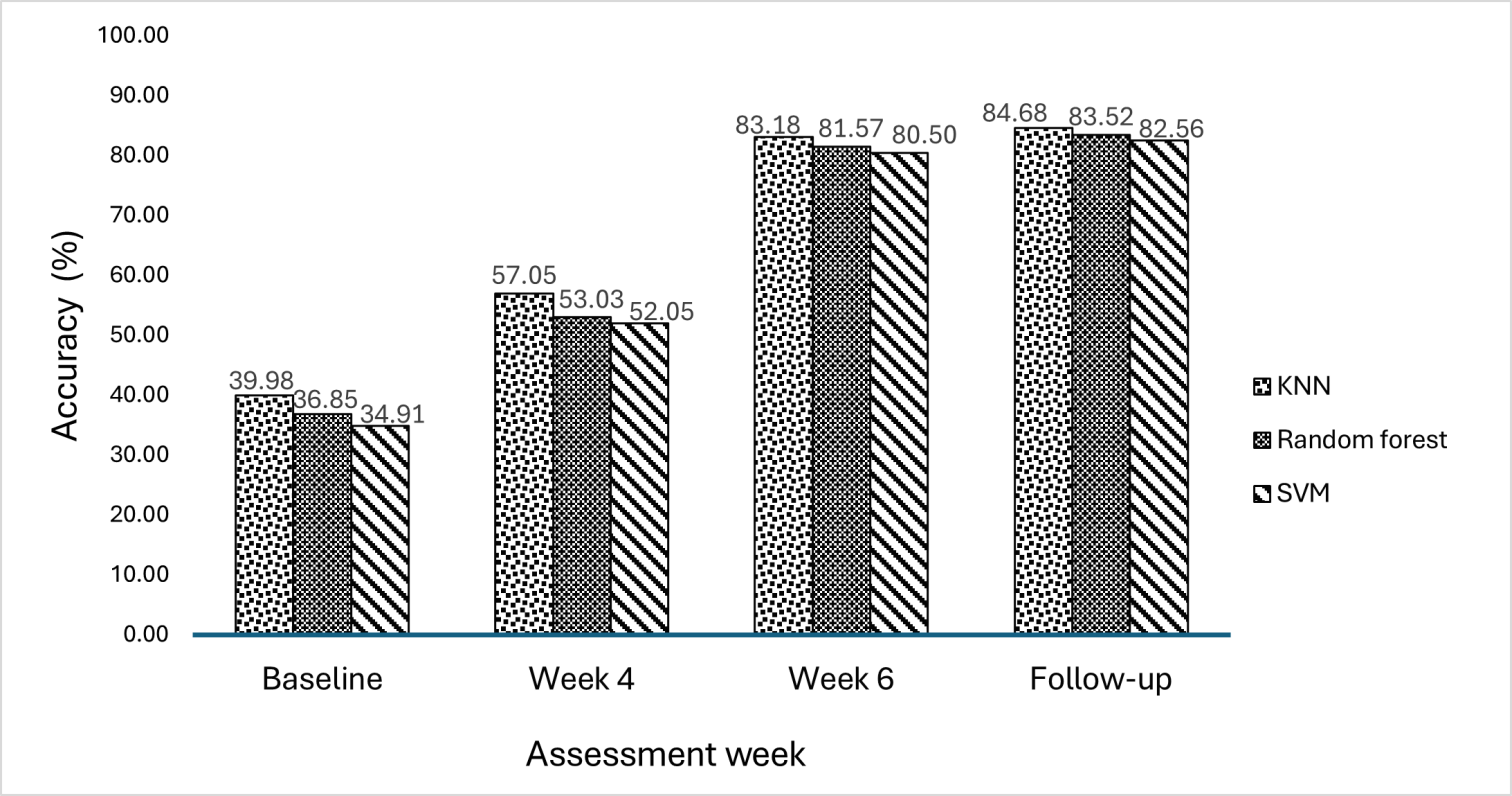
Classification accuracies in the experimental group. KNN: k-nearest neighbor; SVM: support vector machine.

The progressions of classification accuracies (KNN, RF, and SVM) obtained from EMG signal features are depicted in Figures12  13. These accuracies were evaluated at baseline, week 4, week 6, and week 9 (follow-up) assessments in both the experimental and control groups. For KNN, RF, and SVM, the experimental group had baseline accuracies of 39.98%, 36.85%, and 34.91%, respectively, while the control group had baseline accuracies of 40.05%, 37.28%, and 35.15%, respectively. Due to antagonist co-contraction, reduced selective muscle activation, and excessive variability, these low values revealed the complications in recognizing movement-specific EMG signals among patients who have experienced a subacute stroke. At week 4, both groups showed some signs of improvement. However, the experimental group showed a marginal increase in movement performance (KNN: 57.05%; RF: 53.03%; SVM: 52.05%) compared to the control group (KNN: 54.85%; RF: 51.63%; SVM: 50.33%). In comparison to traditional training, this implied that VR games produced earlier improvements in neuromuscular consistency and movement performance. At week 6, a notable improvement in movement performance was observed. The experimental group exhibited higher classification accuracies for movement performance (KNN: 83.18%; RF: 81.57%; SVM: 80.50%), while the control group plateaued (KNN: 77.95%; RF: 77.78%; SVM: 76.32%). This notable improvement in movement performance in the experimental group indicated that patients receiving VR games with enhanced visual feedback showed improved dexterity and voluntary motor control. During the follow-up, the experimental group exhibited sustained high accuracies (KNN: 84.68%; RF: 83.52%; SVM: 82.56%), indicating the strength of their recovery, while the control group plateaued at lower levels (KNN: 79.64%; RF: 79.72%; SVM: 78.25%). This indicates that VR games with enhanced visual feedback effectively sustain hand motor function.

**Figure 13.**
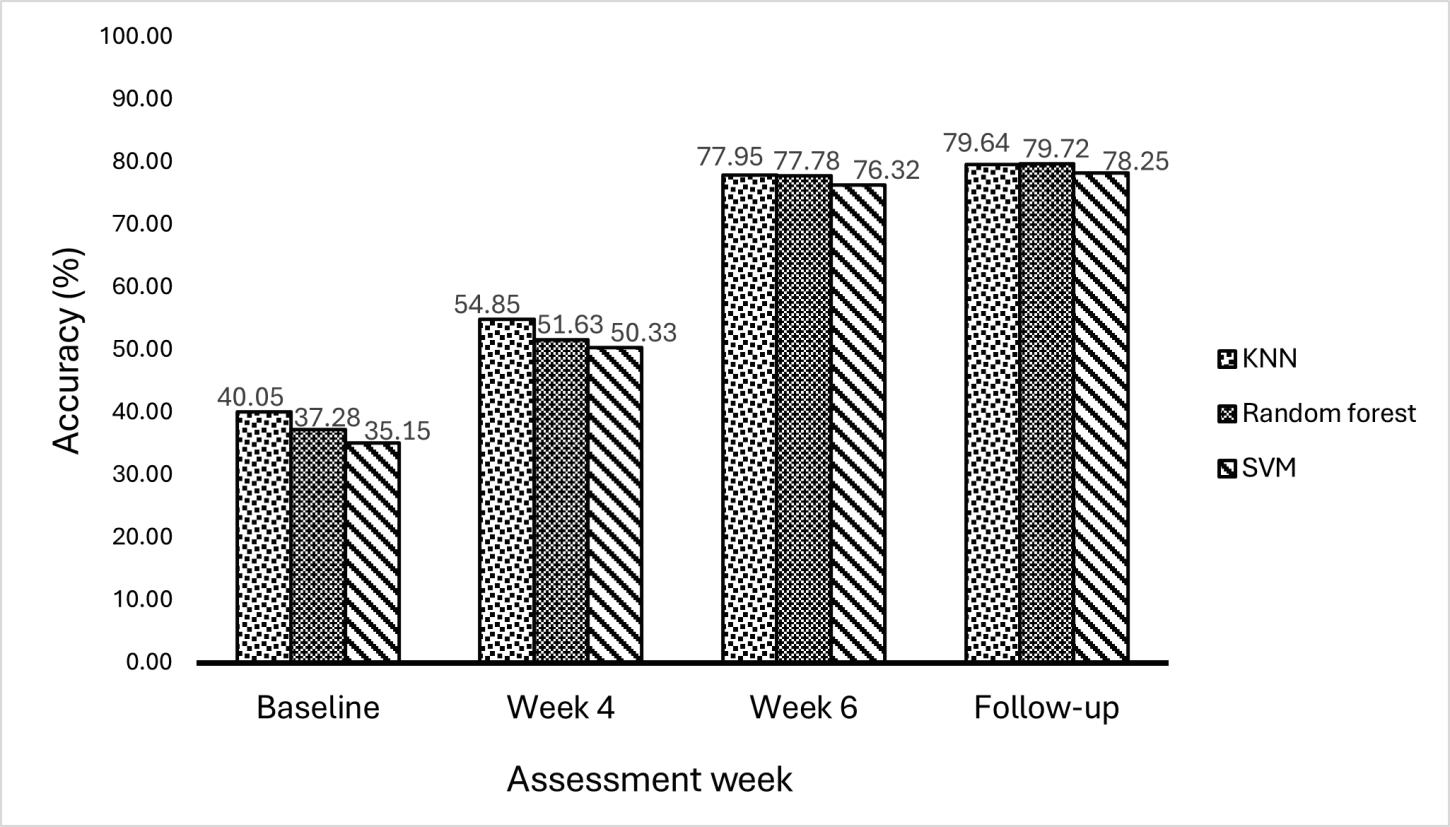
Classification accuracies in the control group. KNN: k-nearest neighbor; SVM: support vector machine.

The results in Figure 12 indicate that the experimental group had better improvements in motor functional abilities due to better progression in weekly movement performance. The weekly accuracy increased in all classifiers; however, the highest movement classification accuracy of 84.68% was obtained in the KNN classifier. As shown in Figure 13, the control group also progressed in weekly movement performance, with the highest movement classification accuracy of 79.72% in the RF classifier. Comparing the movement classification accuracies in both groups, it was found that the experimental group had higher weekly movement classification accuracies than the control group. This implied that the functional abilities of the hand had been effectively promoted through the VR-based game intervention in the experimental group.

**Figure 14.**
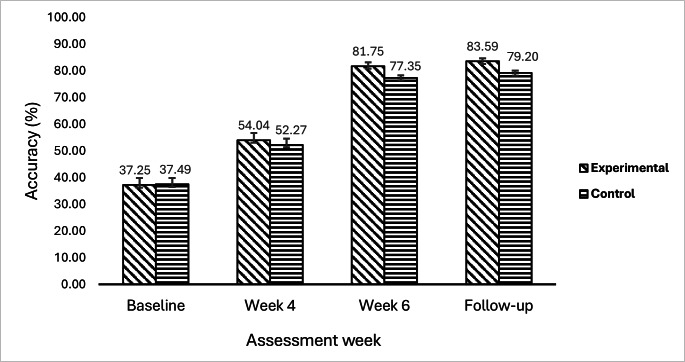
Mean classification accuracies of classifiers in the experimental and control groups. The error bars show the SD.

The mean classification accuracies of the 3 classifiers (KNN, RF, and SVM) are shown in Figure 14. These accuracies are presented for both the experimental group and the control group across all evaluation weeks (baseline, week 4, week 6, and follow-up). The mean accuracies of the 2 groups were nearly identical at baseline (experimental group: 37.24%, SD 2.6%; control group: 37.49%, SD 2.4%), indicating that patients commenced the study at a comparable degree of impairment with weak EMG signal separability. At week 4, both groups exhibited modest improvements in movement performance, with the experimental group experiencing slightly greater improvements in terms of accuracy (mean 54.04%, SD 2.6%) compared to the control group (mean 52.27%, SD 2.3%). This revealed that EMG signals showed slight improvements in movement performance due to the use of VR games in the experimental group. At week 6, the experimental group achieved a higher accuracy (mean 81.75%, SD 1.3%) compared to the control group (mean 77.35%, SD 0.9%). This indicated that EMG signals showed increased improvements in movement performance because of the effective use of VR-based hand games for enhancing hand motor function in the experimental group. At follow-up, the mean movement accuracy was notably higher in the experimental group than in the control group (mean 83.59%, SD 1.1% vs mean 79.20%, SD 0.8%) (Figure 14). This revealed that the hand motor function of patients with subacute stroke was effectively sustained through the use of the VR-based intervention in the experimental group.

## Discussion

### Impact of Enhanced Visual Feedback on Hand Motor Recovery

This research assessed the effectiveness of customized VR-based hand rehabilitation games with an innovative approach of integrating cognitive engagement within visual training feedback. Patients with subacute stroke performed activities in the VR-based hand games that corresponded to their functional tasks. For instance, the game “hit a rolling ball” involved extending and grabbing, the game “grasp a balloon” involved holding and releasing, the game “swap hands” imitated forearm rotation, and the game “grip a pencil” simulated the activities that necessitate fine motor control. These tasks were selected to perceive the achievability of game design contents in real-life scenarios. They were adopted owing to their applicability to movements that occur in real-world environments. The patients with subacute stroke who performed the activities exhibited promising outcomes and improved hand motor function, including increased dexterity, improved ROM, increased hand strength and grip, and enhanced quality of life. The cognitive-sensory paradigm provides an effective rehabilitation approach for long-lasting improvements in hand motor function. In addition to motivating patients, this approach improves the motor learning process to execute hand movements for performing tasks and supports cognitive engagement for undertaking these tasks. This innovative approach to gamification enhances neuroplasticity, which subsequently causes long-lasting improvements in motor function. Moreover, enhanced visual training in VR games involves activities that enhance spatial perception. Many daily tasks require this skill, and it may be particularly important for people undergoing hand rehabilitation.

Most previous studies [43,46-49] applied machine learning algorithms to EMG signals for pattern recognition and gesture control–based applications such as prosthetic control. However, longitudinal rehabilitation assessments lack the use of machine learning algorithms and clinical measures in correlation with EMG signals. Although most previous research [13,16,19,20,44] assessed rehabilitation before and after interventions, there was a neglect of longitudinal assessments to effectively monitor and evaluate enduring improvements in patients with subacute stroke. Therefore, it is considered necessary to develop VR-based hand rehabilitation games for achieving enduring improvements in hand motor function and to conduct longitudinal assessments for monitoring movement performance. A previous study investigated the subjective assessment of the effectiveness of developed VR-based games based on clinical outcome measures (FMA-UE, ARAT, and BBT) [26], and the results showed significant improvements in hand motor function.

### Improvements in Clinical Outcome Measures and the MCID

According to the results in Table 2, the mean difference in the FMA-UE score between the experimental and control groups exceeded the FMA-UE MCID (9.46 > MCID_FMA-UE_). This finding reveals that the experimental group had clinically meaningful improvements in their motor recovery compared to the control group. Thus, the fully immersive VR-based game intervention with enhanced visual training had a positive effect on motor recovery with substantial improvements in the experimental group.

Moreover, the mean difference in the ARAT score between the 2 groups was greater than the ARAT MCID (8.96 > MCID_ARAT_). This finding implies that the experimental group had clinically substantial improvements in their motor recovery due to the intervention involving VR games with enhanced visual training for promoting functional abilities.

Furthermore, the mean difference in the BBT score between the 2 groups exceeded the BBT MCID (20.46 > MCID_BBT_). This finding indicates that the experimental group experienced clinically substantial improvements in hand dexterity due to the task-specific and repetitive VR-based pinch game with enhanced visual training.

### Correlations Between EMG Signal Features and Clinical Outcome Measures

At baseline, there was a weak and nonsignificant (*P*>.05) relationship between muscle activation and hand motor performance in both groups. This finding implies that the muscles had limited strength before the intervention and were not effective in performing hand functional tasks due to motor control impairments. In the early phases of stroke recovery, patients may have ineffective activation of muscle patterns due to neurological and motor control impairments. Weak correlations between EMG features and clinical outcome measures may result from ineffective muscle activation, which may not accurately indicate the functional capacity of the damaged limb.

From Figures 6-11, at week 6, both the experimental and control groups showed positive and significant (*P*<.05) correlations between EMG signal features and clinical outcome measures. However, the experimental group showed strong positive correlations, while the control group showed moderate positive correlations. The strong positive correlations in the experimental group indicate significant improvements in hand motor function, coordination, hand dexterity, and fine grip strength. These improvements reflect higher FMA, ARAT, and BBT scores with increased EMG signal features. The strong correlations in the experimental group illustrate the effectiveness of cognitive engagement within visual feedback incorporated into VR-based games that significantly improved hand motor function. Furthermore, the VR-based games were repetitive and engaging, enhancing hand motor function recovery in the experimental group. At week 6, the control group demonstrated moderate positive correlations. This implies that improvements in the control group were less substantial than those in the experimental group. Thus, conventional physical therapy alone was beneficial but was not as effective as the approach involving VR-based game therapy used in the experimental group, which enhanced the motor recovery of hand function.

During the follow-up at week 9, there were significant (*P*<.05) strong and moderate positive correlations in the experimental and control groups, respectively. These results imply that both groups experienced improvements in motor recovery, but the experimental group showed better sustained motor recovery than the control group, indicating a strong association between EMG signal features and improvement in hand motor function.

Results between baseline and follow-up showed notably substantial improvements, with the experimental group showing a change from nonsignificant (*P*>.05) weak positive correlations to significant (*P*<.05) strong positive correlations and the control group showing a change from nonsignificant (*P*>.05) weak positive correlations to significant (*P*<.05) moderate positive correlations. These findings indicate that the intervention of VR-based games in the experimental group effectively sustained hand motor function, coordination, hand dexterity, and fine grip strength among patients with subacute stroke. VR games that target hand movements can strengthen relevant muscles, and these benefits promote functional gains in hand motor tasks over time. The longitudinal assessment of correlations provides insights into muscle activation and clinical outcome measures in VR game rehabilitation for better evaluation of hand motor function over time to achieve enduring improvements.

### Impact of EMG-Based Progression Analysis of Movement Performance

EMG-based classification accuracies were used to evaluate movement performance in assessment weeks for the effectiveness of VR-based hand rehabilitation games. This provided information on the relationship between changes in EMG characteristics and improvements in motor skills. The results in Table 3 revealed significant differences between the experimental and control groups in all classifiers (all *P*<.05). At baseline, the mean movement classification accuracies in the experimental and control groups were 37.24% (SD 2.6%) and 37.49% (SD 2.4%), respectively. These low accuracies were due to decreased muscle activity patterns in the EMG signals associated with different hand movements.

Figure 12 shows that movement accuracy increased as rehabilitation progressed in subsequent weeks, including weeks 4, 6, and 9 (follow-up). This suggests that advances in hand movements and motor activities were being recognized by EMG features, which were becoming increasingly distinct and reliable. The experimental group exhibited higher classification accuracies in weeks 4, 6, and 9 (follow-up) compared to the control group, as illustrated in Figure 12. This shows that due to challenging levels and cognitive engagement within visual feedback, the experimental group adapted to more functional engagement in VR-based games in subsequent weeks. This increased muscle activity patterns in EMG signals, which improved the motor function of the hand in the experimental group. The control group also showed higher movement classification accuracy over time, as shown in Figure 13. However, the accuracy was lower than that in the experimental group.

In the experimental group, the KNN, RF, and SVM models achieved high movement accuracies, with rates of 84.68%, 83.52%, and 82.56%, respectively. This indicates that patients’ motor function improvements were significant owing to the VR-based game intervention, and the approach effectively enhanced muscle control. The greater accuracy in the experimental group highlights the long-term advantages of VR-based rehabilitation. This implies that EMG features are reliable indicators of motor function improvements. The combined accuracies of all classifiers at follow-up, as presented in Figure 14, show that the experimental group had a higher mean accuracy (83.59%, SD 1.1%) than that in the control group (79.20%, SD 0.8%). This reveals that the VR-based hand games efficiently promoted repeated muscle activations, resulting in improved hand motor function, which was sustained in the experimental group. At baseline, there were no significant differences between the groups (*P*>.05), indicating equivalent starting points. The difference in machine learning prediction accuracy between the 2 groups was statistically significant (*P*<.05) after week 2, with the experimental group demonstrating consistently superior performance by weeks 4 and 6. The notable differences between the groups demonstrate that the VR-based training not only expedited motor recovery but also generated more distinct, stable, and classifiable EMG activation patterns, which enhanced machine learning prediction reliability. This suggests that immersive VR is more effective in improving the neurophysiological foundations of motor control compared to traditional therapy alone.

VR training improved the quality of EMG signals, as evidenced by the higher accuracies of all 3 classifiers in the experimental group at weeks 4, 6, and 9, by decreasing intraclass variability (making signals for the same movement more consistent) and increasing interclass separation (making different movements more distinct). KNN performed better than RF and SVM, indicating that VR training produced compact, well-clustered EMG feature distributions, where a distance-based approach was adequate for accurate classification. Following VR training, RF and SVM exhibited slightly weaker performance, suggesting that complicated decision boundaries were no longer necessary to achieve high separability in the EMG data. The fact that KNN performed the best indicates that the EMG signals of the patients had become naturally separable, which suggests that their motor recovery proceeded toward more predictable and distinct muscle commands. Conventional therapy only slightly improved the control group’s EMG data, which continued to exhibit greater variability and class overlap. The absence of differences among KNN, RF, and SVM classifiers was not able to completely overcome the signal inconsistency, indicating that patients still lacked precise neuromuscular recovery. Patients in the control group did not achieve the same degree of consistent, distinct EMG activation patterns, as noted in the accuracy values. Their motor intent was less predictable because their muscle activity continued to overlap.

Within VR gaming, visual feedback helps maximize the performance of patients with stroke, ultimately contributing to their motion adaptation and improvement. Using a VR headset with hand tracking, patients were able to actively participate in the games while simultaneously viewing their damaged hands. Owing to this advantage, patients could easily move and control the affected hand.

### Implications for Stroke Rehabilitation

This study offers several implications for stroke rehabilitation that may inform accessible practice and research on stroke rehabilitation support, suggesting a new VR-based game approach. The integration of EMG features as a quantitative method facilitates the objective monitoring of patient development, potentially reducing dependence on subjective scales alone. The gamified VR tasks enhanced functional engagement and motivation, which have been recognized as obstacles in traditional stroke recovery [3]. The integration of gameplay challenges and increased visual feedback in immersive VR rehabilitation games enhanced compliance and sustained long-term improvements in hand motor function. Furthermore, through the involvement of interactive and task-oriented training, the VR games led to improved functional performance. This signifies that these VR rehabilitation games can serve as valuable complements to conventional care, particularly for patients with reduced motivation or those who have restricted access to supervised therapy sessions. Moreover, a framework has been established for objective recovery monitoring by merging EMG with machine learning.

The findings of this research enable the integration of innovative sensory-cognitive engagement and functionally challenging activities with targeted movements. This contributes to effective improvements in hand motor function and enhances neuroplasticity in the brain. Moreover, in patients with subacute stroke, a gamified setup with sensory-cognitive engagement provides intrinsic motivation to engage actively during VR sessions. From easy to difficult levels, patients adapt to the challenges of motor task activities, which can enhance their involvement and help promote effective recovery of hand motor function. The outcomes obtained from this research underscore the correlations between EMG signals and clinical outcomes over time and the efficacy of a machine learning–based approach involving weekly progression for evaluating hand motor function.

### Future Directions

In the future, a complete pipeline will be developed in Unity or C# that can facilitate seamless integration with a VR device. Moreover, the modular architecture offers a scalable framework that can be integrated into a singular system environment in future versions. The VR rehabilitation games implemented in this study have the potential to be more broadly applied across impairment profiles, including activities that require fine-motor dexterity and bilateral coordination.

### Limitations

This study has some limitations. First, the VR games were confined to a limited number of exercises. Some individual stroke survivors could require specific exercises for their conditions. Second, blinding in this study was not feasible as the patients and therapists were informed of the intervention. To minimize potential outcome bias, standardized intervention protocols and consistent instructions were used across the groups. Furthermore, the incorporation of objective EMG-based outcome measures and machine learning–derived movement performance classification accuracies facilitated an unbiased evaluation of motor recovery, thus reducing the possible influence of this constraint. Third, the evaluators in this study were not blinded. Therefore, further objective assessment may be necessary for validation. Fourth, supplementary clinical outcome measures were not included. It may be necessary to consider specific supplementary clinical outcome measures to further evaluate VR-based hand activities.

### Conclusion

This study evaluated the effectiveness of immersive VR-based hand games in patients with subacute stroke, using clinical outcome measures (FMA-UE, ARAT, and BBT) and EMG-based features. Significant differences were observed in all clinical outcome measures (all *P*<.05) between the experimental and control groups. The experimental group experienced clinically meaningful improvements, with mean differences in FMA-UE, ARAT, and BBT scores being greater than their MCIDs. Furthermore, the results demonstrate the effectiveness of a machine learning approach involving weekly progression for performance assessment. The better follow-up correlation results (positive, strong, and significant) between EMG features and clinical outcome measures and the higher movement performance accuracies in the experimental group than in the control group indicate that the VR-based hand games were effective and helped sustain motor function in patients with subacute stroke. The findings of this study reveal that conventional therapy combined with immersive VR-based rehabilitation games involving enhanced visual training feedback can enhance motor recovery and movement performance more effectively than conventional therapy alone.

## Supplementary material

10.2196/74314 Multimedia Appendix 1 Visuals of the virtual reality–based rehabilitation games.

10.2196/74314 Checklist 1 CONSORT-EHEALTH checklist.

## Abbreviations

ARAT: Action Research Arm Test
BBT: Box and Block Test
CONSORT: Consolidated Standards of Reporting Trials
EMG: electromyography
FMA-UE: Fugl-Meyer Assessment-upper extremity
KNN: k-nearest neighbor
MCID: minimal clinically meaningful difference
RF: random forest
ROM: range of motion
SDK: software development kit
SVM: support vector machine
VR: virtual reality
