# Supplementary material for: Evaluating the Effectiveness of Immersive Virtual Reality Rehabilitation Games With Enhanced Visual Training for Hand Motor Function Improvement Using Electromyography: Randomized Controlled Trial
Source: JMIR Serious Games. 2025 Nov 25;13:e74314. doi: 10.2196/74314 (PMC12646558; doi:10.2196/74314)
Supplement: Multimedia Appendix 1 [file games-v13-e74314-s001.pdf]

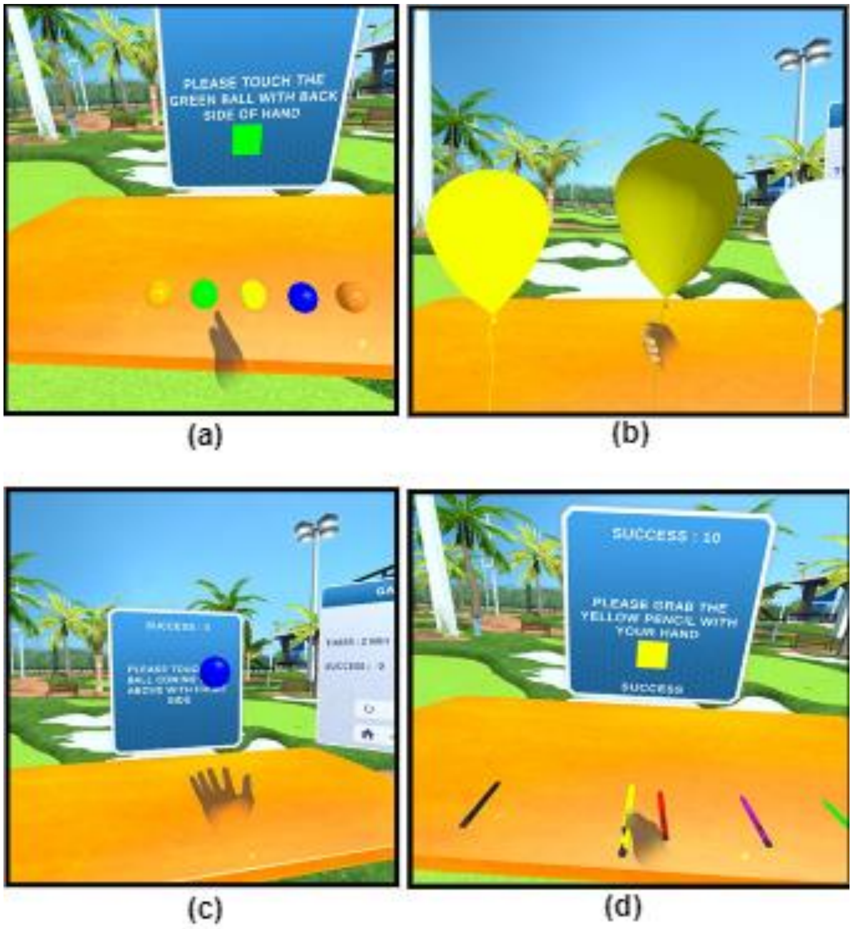

VR game rehabilitation visuals: (a) Hit a rolling ball, (b) Grasp a balloon, (c) Swap hands, and (d) Grip a pencil
